# Cardiomyocyte Imaging Using *Real-Time* Spatial Light Interference Microscopy (SLIM)

**DOI:** 10.1371/journal.pone.0056930

**Published:** 2013-02-15

**Authors:** Basanta Bhaduri, David Wickland, Ru Wang, Vincent Chan, Rashid Bashir, Gabriel Popescu

**Affiliations:** 1 Department of Electrical and Computer Engineering, University of Illinois at Urbana-Champaign, Urbana, Illinois, United States of America; 2 Department of Bioengineering, University of Illinois at Urbana-Champaign, Urbana, Illinois, United States of America; University of Campinas, Brazil

## Abstract

Spatial light interference microscopy (SLIM) is a highly sensitive quantitative phase imaging method, which is capable of unprecedented structure studies in biology and beyond. In addition to the π/2 shift introduced in phase contrast between the scattered and unscattered light from the sample, 4 phase shifts are generated in SLIM, by increments of π/2 using a reflective liquid crystal phase modulator (LCPM). As 4 phase shifted images are required to produce a quantitative phase image, the switching speed of the LCPM and the acquisition rate of the camera limit the acquisition rate and, thus, SLIM's applicability to highly dynamic samples. In this paper we present a fast SLIM setup which can image at a maximum rate of 50 frames per second and provide in real-time quantitative phase images at 50/4 = 12.5 frames per second. We use a fast LCPM for phase shifting and a fast scientific-grade complementary metal oxide semiconductor (sCMOS) camera (Andor) for imaging. We present the dispersion relation, i.e. decay rate vs. spatial mode, associated with dynamic beating cardiomyocyte cells from the quantitative phase images obtained with the real-time SLIM system.

## Introduction

Quantitative phase imaging (QPI) has emerged as a highly sensitive method for measuring nanometer scale pathlength changes induced by a specimen [1]. This *optical* information yields *biological* information, including cell mass [2], [3], [4], [5], membrane fluctuations [6], [7], cell tomography [8], [9], [10], [11], intracellular transport [12], [13], tissue scattering [14], [15], [16], blood testing [17], [18], [19], cancer diagnosis [20]. Recently, a number of QPI methods have been developed for such biomedical applications [21], [22], [23], [24], [25], [26], [27], [28], [29], [30]. However, the contrast in QPI images has always been degraded by speckles resulting from using highly coherent light sources such as lasers. The spatial non-uniformity caused by speckles is due to the random interference phenomenon caused by the coherent superposition of various fields scattered from the specimen, optical surfaces, imperfections, or dirt. This superposition of fields yields a more uniform background if the pathlength difference between the fields is less than the coherence length (*l_c_*) of the light. Thus, using broadband fields increases the spatial sensitivity of QPI (see, e.g., Chapter 8 in Ref. [1]).

Spatial light interference microscopy (SLIM) is such a highly sensitive QPI method, which enables new studies in biology and beyond [9], [31]. SLIM combines two classical ideas in optics and microscopy: Zernike's phase contrast method [32], by revealing the intrinsic contrast of transparent samples, and Gabor's holography [33], by quantitatively retrieving the phase information. SLIM provides the spatial uniformity associated with white light methods and the stability associated with common path interferometry. In addition, due to the short coherence length of the illumination, SLIM also provides excellent optical sectioning, enabling three dimensional tomography [9]. In SLIM, besides the π/2 shift introduced in phase contrast between the scattered and unscattered light from the sample, additional 3 phase shifts are generated. These shifts are performed in increments of π/2 using a reflective liquid crystal phase modulator (LCPM). As 4 phase shifted images are required to produce a quantitative phase image, the switching speed of the LCPM and the acquisition rate of the camera limit the acquisition rate and, thus, SLIM's applicability to highly dynamic samples.

In this paper we present a fast SLIM setup which can image at a maximum rate of 50 frames per second and provide in real time quantitative phase images at 50/4 = 12.5 frames per second. This performance was achieved by combining a fast LCPM (Boulder Nonlinear Systems) and a fast scientific-grade complementary metal oxide semiconductor (sCMOS) camera (Andor Neo). Further, using novel software developed in house, we perform the phase reconstruction and display the quantitative phase images in real time.

Furthermore, we used dispersion-relation phase spectroscopy (DPS) to study intracellular mass transport in beating cardiomyocytes. DPS is an analysis modality that uses the time-lapse QPI data to quantify mass transport in continuous and transparent systems [34], [35]. The DPS approach is significantly faster than traditional methods for studying transport, as it does not require tracking individual particles. DPS also applies to particles which are smaller than the diffraction spot of the microscope, i.e., particles that are not resolved by the imaging system. In living cells where there are usually not many intrinsic particles available for tracking, DPS provides an efficient alternative to adding extrinsic particles to cells. Since DPS uses SLIM to acquire the phase maps, the total dry mass of the cell and other information such as fluorescence may be acquired simultaneously. Using DPS, several cell types have been studied in the past, including neurons, glial, and microglial cells [34], [35]. Here we present the dispersion relation, i.e. decay rate vs. spatial mode, associated with beating cardiomyocyte cells. The quantitative phase images obtained with the real-time SLIM system provide the necessary time resolution for understanding mass transport at the fast scales involved in beating cardiomyocytes. Remarkably we found that this transport follows the pattern of a deterministic phenomenon, as one might expect from a pulsating cell.

## Methods

### Real-time Spatial light interference microscopy

**Figure 1:**
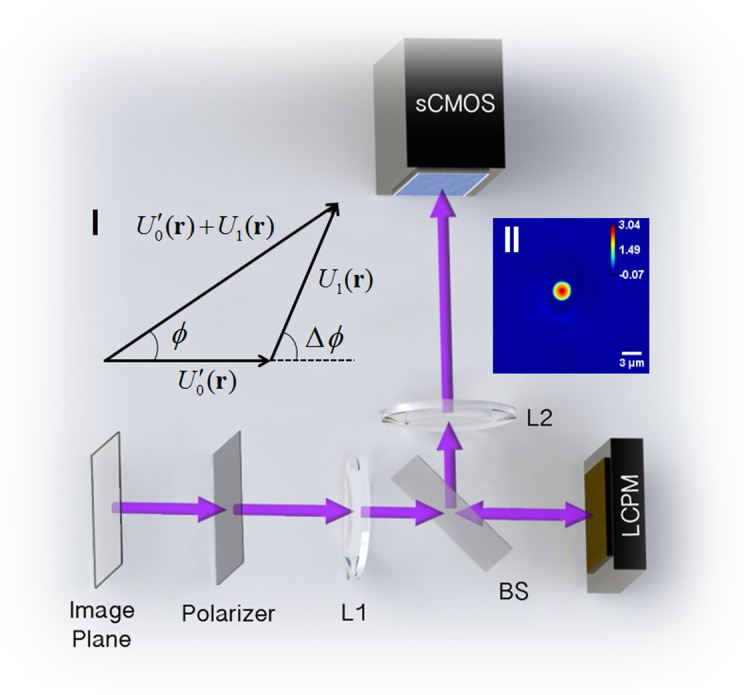
Real-time SLIM setup. The inset I shows the coherent summation of the scattered and unscattered lights. The inset II shows the SLIM image of a micro-bead (3.0 µm diameter) immersed in oil. The measured height is 3.04 µm.

A schematic of the SLIM experimental setup is shown in Fig. 1. The imaging system represents a 4f, *telecentric* system, characterized by the fact that an ensemble of parallel rays at the input plane remains parallel at the output plane. SLIM is designed as an add-on module to a commercial phase contrast microscope (Axio Observer Z1, Zeiss). As in phase contrast microscopy, SLIM relies on the spatial decomposition of the image field into its scattered and unscattered components. The concept of image formation as the interference between these two components is fundamental to understanding SLIM's principle [9], [31]. In addition to the conventional π/2 shift introduced between these two light components, we introduced further phase shifts in increments of π/2. This additional modulation was achieved by using a reflective LCPM (Boulder Nonlinear Systems). The LCPM is placed in the Fourier plane of this system which is conjugate to the back focal plane of the microscope objective (Zeiss, 40X, Ph 2, NA = 0.75) which contains the phase contrast ring. The active pattern on the LCPM is calculated to precisely match the size and position of the phase contrast ring image, such that additional phase delay between the scattered and unscattered components of the image field can be controlled. In this setup, four images (*I*_m_, m = 1, 2, 3, 4) corresponding to each phase shift, (m-2)π/2, are recorded sequentially, to produce a quantitative phase image that is uniquely determined. The LPCM is calibrated for phase shifting as detailed elsewhere [31]. The polarizer P ensures the LPCM is operating in a phase modulation only mode. The lens L_1_ has a focal length (f_1_) of 150 mm where L_2_ has a focal length (f_2_) of 200 mm, thus we get further f_2_/f_1_ magnification outside the microscope. Further we have used a scientific-grade complementary metal oxide semiconductor (sCMOS) camera (Andor) for imaging. This 5.5 Mega Pixel camera has smaller pixels that improve resolution by preserving Nyquist sampling and capable of imaging at 100 frames/s at full frame [36]. sCMOS has been expected to be used in a wide range of application fields which are currently dominated by Electron-Multiplication Charge Coupled Device (EMCCD) [37].

Inset I in Fig. 1 illustrates the coherent summation of the scattered, 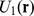, and unscattered, 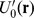 fields, where 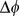 is the spatially varying phase shift between 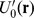 and 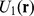, and 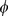 is the phase associated with the image field (the quantity of interest in QPI). Due to the phase contrast ring, we measure an attenuated unscattered field, 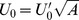, where 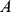 is the intensity attenuation provided by the ring in the objective. The phase 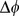 can be expressed as:
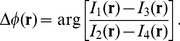
(1)


Letting 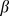 as the ratio of the scattered and unscattered field amplitudes, 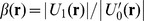, the phase associated with the image field is determined as [31]:
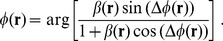
(2)


The factor 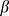 can be obtained by solving the equation:
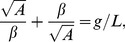
(3)


where
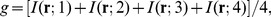
(4a)




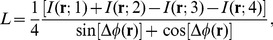
(4b)and *I*(r, m); m = 1..4, are the four measured intensity images.

Note that Eq. 3 is symmetric in terms of 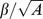 and 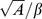, therefore the two solutions are 
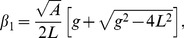
(5a)

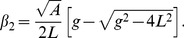
(5b)


However, in the limit 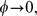
the physically valid solution for 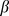 is 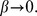 Thus the relevant value of 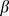 can be obtained by evaluating the expression on the right-hand side of Eq. 3 at point 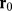, where it has small value, and setting 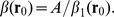 The quantity 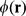 is therefore uniquely determined from the four interferograms with no additional measurements.

### Sample preparation

Cardiomyocytes were obtained as previously described [38] using an approved protocol by the University of Illinois at Urbana-Champaign (UIUC) Institutional Animal Care and Use Committee (IACUC; Protocol #11160). Briefly, whole hearts from 0-2 day old neonatal Sprague-Dawley rats (Harlan Laboratories) were excised and placed in ice-cold HBSS buffer [39]. Using small scissors, the left and right atria were removed and the remaining ventricles were quartered. The quartered ventricles were digested in 0.05% (w/v) purified trypsin (Worthington Biochemicals Corp.), while rotating gently at 4°C overnight. After 18 hours, warm growth medium was added for 5 minutes at 37°C to inhibit trypsin digestion. After washing and discarding the supernatant, 0.1% (w/v) purified type II collagenase (Worthington Biochemicals) was added for 45 minutes while rotating at 37°C. The tissue was gently triturated to mechanically loosen the cells, and the suspension was filtered through a 40 µm cell strainer. The suspension was removed after centrifugation at 150 x g for 6 minutes. The remaining cell pellet was re-suspended in warm growth medium and pre-plated twice for 30 minutes each to enrich for cardiomyocytes. The suspension was collected, and cardiomyocytes were seeded on polystyrene dishes. The cells were cultured on rat tail type I collagen-coated (BD Biosciences) glass-bottom dishes (MatTek) in an incubator with 5% CO_2_ at 37°C. The growth medium consisted of high glucose Dulbecco's modified Eagle's medium (DMEM) with 2.5% fetal bovine serum (FBS) and 10% horse serum (HS).

### Dispersion-relation phase spectroscopy (DPS)

Dispersion-relation phase spectroscopy (DPS) provides the ability to quantify mass transport in continuous and transparent systems in a label free manner [34], [35]. Experiments on live cells using this method have shown that the transport is diffusive at scales below a micron and deterministic at larger scales as expected from current knowledge about biology. As the SLIM image may be regarded as a 2D dry mass density map [31] and thus the changes in this map satisfy an advection-diffusion equation that includes contributions from both directed and diffusive transport [35]: 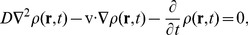
(6)


where *D* is diffusion coefficient, **v** is the advection velocity and 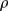 is the dry mass density. In SLIM, the entire forward scattering half space is measured simultaneously, limited only by the numerical aperture of the objective. Thus SLIM essentially functions as a highly sensitive light scattering measurement instrument.

The temporal autocorrelation, *g*, for each spatial mode, *q* is 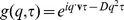, which decays exponentially at a rate Γ, 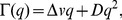
(7)where 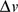 is the bandwidths of the speed distributions.

Equation 7 is the dispersion relationship which gives the technique its name. Thus from a 3D (*x,y,t*) SLIM dataset, the dispersion relationship 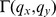
may be calculated by first performing a spatial Fourier transform of each frame and then by calculating the temporal bandwidth at each spatial frequency by performing a temporal Fourier transform. The radial function, 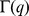, where 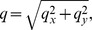 is obtained by an azimuthal average of the data. In practice, once time lapse data set is obtained, 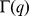
can be calculated and fitted by both linear and quadratic regions to obtain diffusion coefficients *D* and bandwidth of advection velocities, 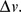


The maximum *q*-value is given by the microscope's resolution, *q*_0_ = 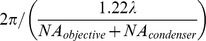. With the current setup, we have 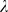 = 0.55 µm (center wavelength),
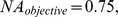
and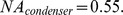 Thus *q*_0_ = 12 rad/µm and data outside this range should be considered as noise.

### Integration and synchronization of the camera and the LCPM

**Figure 2:**
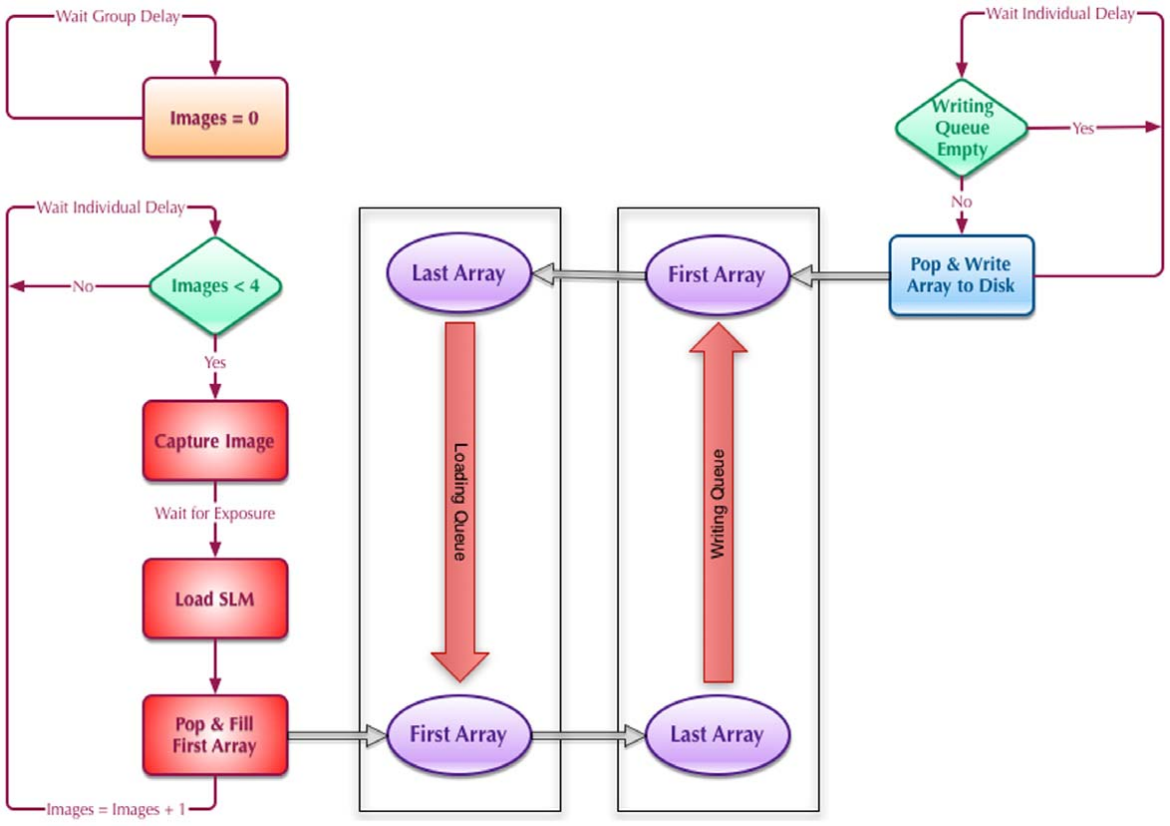
Flowchart of the real-time SLIM software operation.

We have developed software that is capable of capturing the 4 intensity images, processing them and displaying the SLIM images, all in real time. Figure 2 shows the flowchart of the software. This software works on several parallel threads. First, a queue of arrays is allocated to store images as they are captured. Next, the LCPM is loaded with the first phase pattern. Then, two timer threads are created. One is fired every time an individual image is to be taken, while the other fires each time a new group of images begins. The individual timer thread begins by checking how many images in the group have been captured. If all four phase images have not been taken, the thread sends a command to the camera to capture an image. A short wait will ensure the camera has finished capturing before the next phase pattern is loaded onto the LCPM. The captured image is subsequently loaded into the first array popped off the loading queue. Following this, the array is added to another queue for writing the images. The group timer simply resets the capture count so the individual timer thread will capture another four images. While the timer threads are running, the main thread checks for images being loaded in the writing queue. When it discovers one, it writes the image to the disk and adds the array back into the original loading queue. All four phase shifted images are stored in the buffer and before starting the new cycle of phase shifted images, the SLIM image is calculated and displayed in less than 20 ms.

Achieving the goal of real time SLIM imaging at fast rates prompted us to overcome some challenges in data acquisition, as follows. If the sCMOS camera is used in the *global shutter* (snapshot) mode, it is capable of acquiring images at 50 frames/s at full frame resolution (5.5 MP). Though the camera can be used at higher acquisition rates with reduced frame size, the LCPM is the actual component that sets the limit of the acquisition rate as it cannot load the phase rings at more than 50 Hz without introducing errors. Thus, the maximum achievable acquisition rate of intensity images is 50 Hz, which results in 12.5 SLIM images per second. The limited memory of the camera (4 GB), allows us to image only for few seconds at 50 frames/s and 5.5 MP resolution. However, by using a high speed solid state hard drive, we can capture and transfer up to 2 MP resolution images at 50 frames/s for arbitrarily large number of frames. The field of view (with 40× objective) at full frame is around 300×250 µm^2^ which is more than 10 times larger than what we achieved with the previous SLIM implementation [31].

## Results

### Accuracy of the system

In order to assess the accuracy of this new real-time SLIM system, we imaged a 3±0.15 µm polystyrene bead immersed in immersion oil (Zeiss). The inset II of Fig. 1 shows such a SLIM image. The color bar shows the height in µm and the scale bar is in µm. The measured height is 3.04 µm at 0.55 µm (center wavelength of the source) which matches very well the actual diameter.

### Real-time SLIM of cardiomyocytes

**Figure 3:**
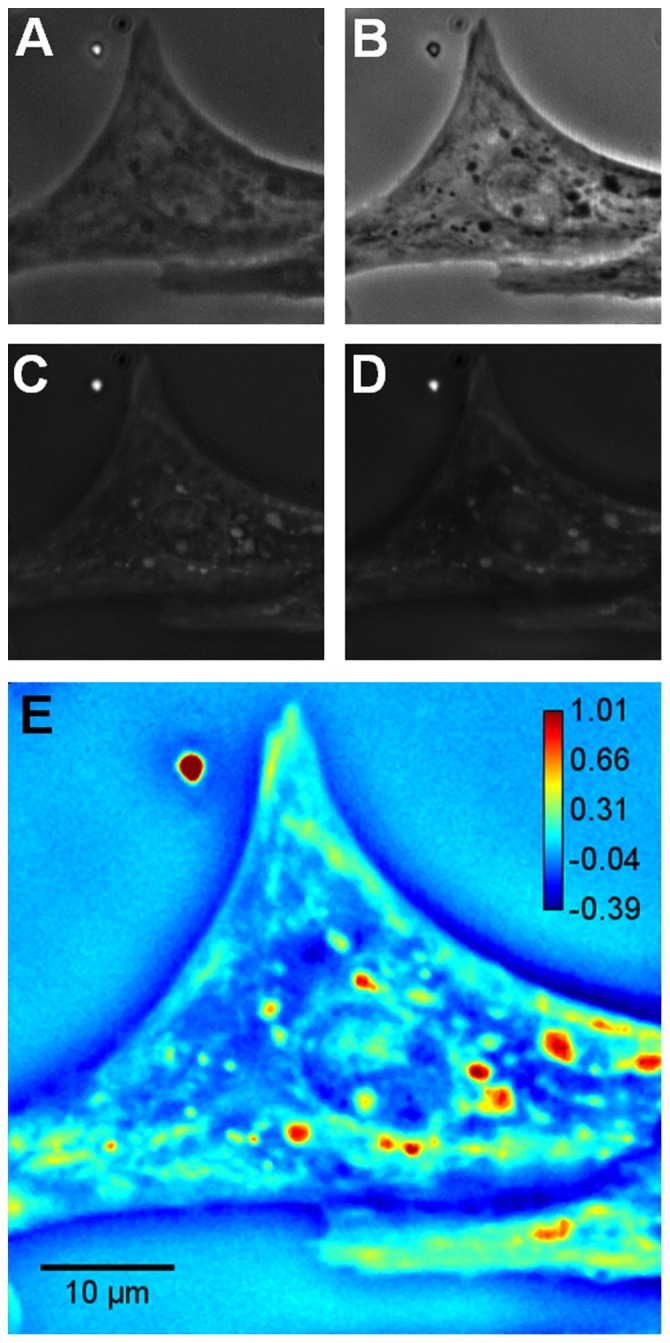
Quantitative phase imaging using real-time SLIM. **A**)**-D**) Intensity images corresponding to the 4 phase shifts for a cardiomyocyte cell. **E**) Resulting SLIM image. The color bar shows the phase in radians.

In order to show the capability of the real-time SLIM for quantitative imaging of dynamic cells, we have imaged beating cardiomyocyte cells. Phase shifted images are captured at 50 frames per second while LCPM changes its patterns at the same rate. Thus the SLIM images are obtained at 12.5 frames per second. Figure 3 shows an example of a SLIM image (Fig. 3E) retrieved in real time by combining the intensity images associated with -π/2, 0, π/2, and π phase shifts (Figs. 3A-D, respectively). Note that Fig. 3B is the conventional phase contrast image.

**Figure 4:**
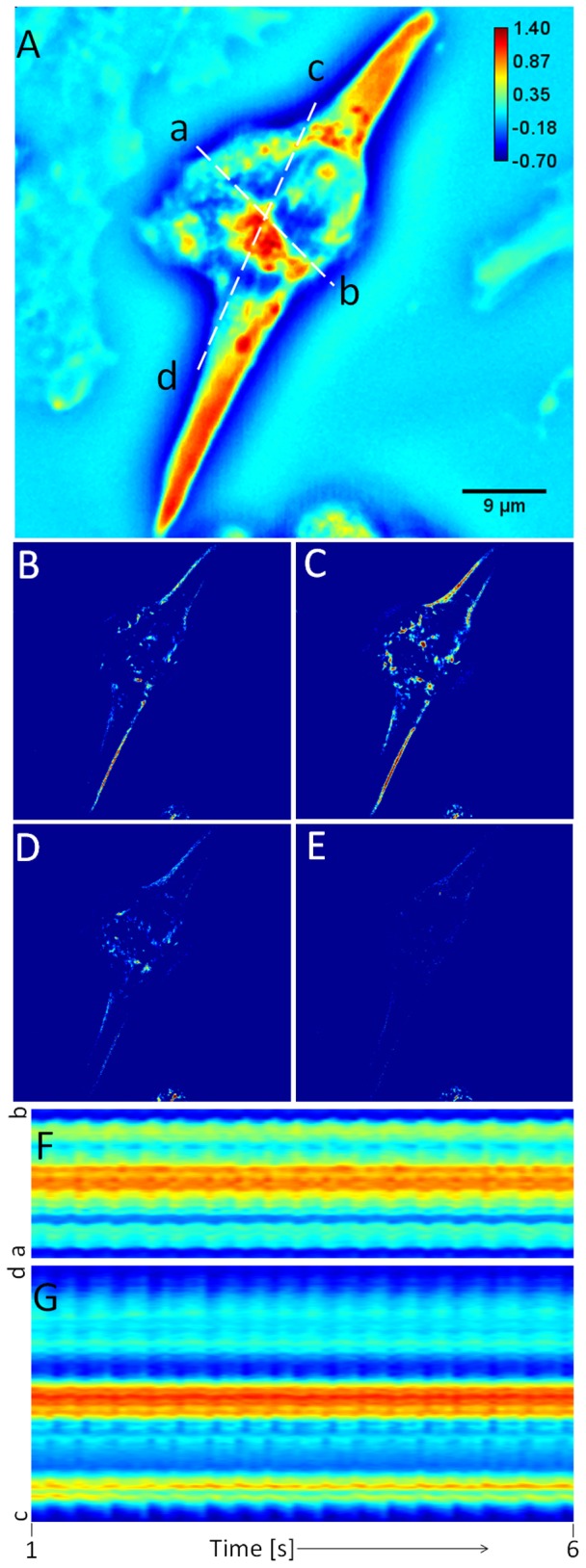
Dynamic cardiomyocyte imaging. **A**) SLIM image of a beating cardiomyocyte cell, **B**)**-E**) Difference of phase images from the initial image during one complete beating cycle, **F**)**-G**) Phase profiles of the cell along ab and cd vs. time. The color bar shows the phase in radians.

Furthermore, to study the sub-cellular movements quantitatively during beating, we acquired time-lapse images of cardiomyocytes beating at frequencies of approximately 2.5 Hz. Again, the SLIM images are obtained at 12.5 frames per second (see Video S1). Figure 4A shows an instantaneous quantitative phase image during the beating of the cell. As the sub-cellular movements are small compared to the size of the cell, the difference of consecutive phase images can illustrate these movements better. Figures 4B-E show the difference of phase images from the initial image during one complete beating cycle. It can be seen from these figures that the cell reaches the peak of displacement (Fig. 4C) and then gradually comes to the rest position (Fig. 4E). To emphasize the ability of SLIM to retrieve spatiotemporal data, we obtained phase profiles along the short (ab) and long (cd) axes of the cell. Thus, Figures 4F-G show pathlength profiles of the cell along ab and cd during beatings. These results indicate that, in principle, one can perform quantitative correlations between various points across the cells or between points on different cells in a population. Here, we aim to understand the physical nature of the mass transport during the beating cycle.

### Dispersion-relation phase spectroscopy (DPS) of cardiomyocytes

**Figure 5:**
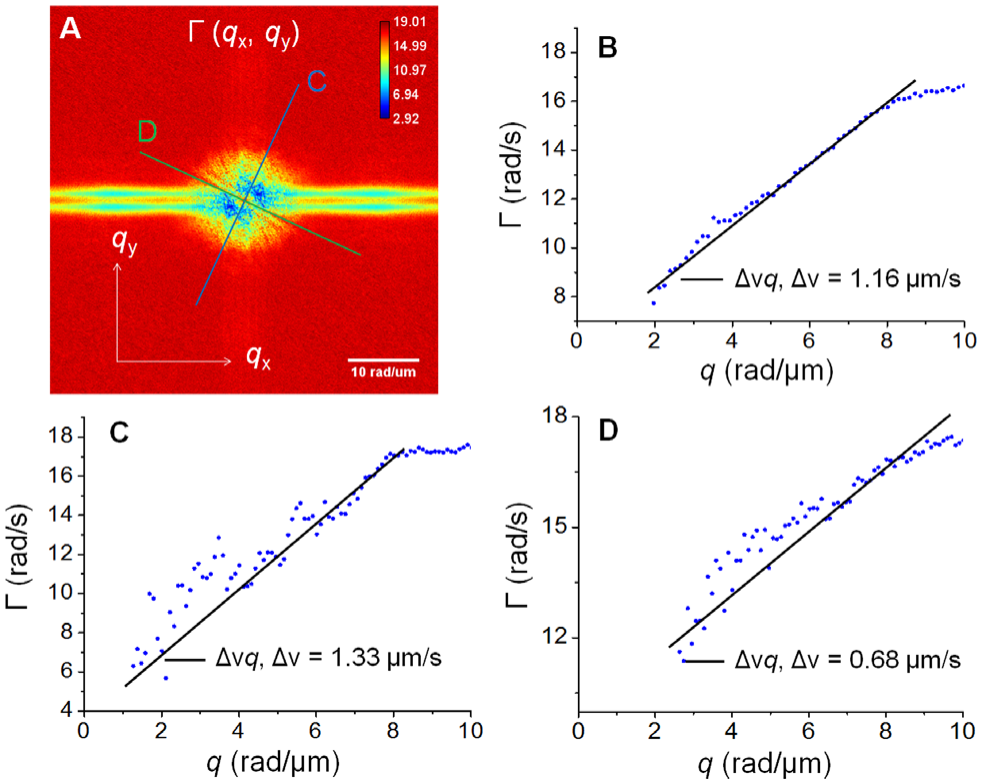
Dispersion-relation phase spectroscopy of cardiomyocyte cell 1. **A**) Dispersion relation map, Γ(*q*) associated with the cardiomyocyte cell shown in Fig. 4, **B**) Profile of the azimuthal average of the data in A, **C**) Angular profile of data in A at 65 degree (blue line in A), **D**) Angular profile of data in A at 155 degree (green line in A). The fit with the linear function yields the value of 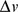 as indicated. The color bar shows Γ values in rad/s.

We measured the dispersion relation, i.e. decay rate vs. spatial mode, associated with beating cardiomyocyte cells. To facilitate calculations, we developed an ImageJ plugin to calculate the dispersion relation map, Γ(*q*). Figure 5A shows such a dispersion-relation map for the cell shown in Fig. 4. Figure 5B shows the profile of the azimuthal average of data in Fig. 5A, assumed to be isotropic. Figures 5C-D show, respectively, the profiles of data at 65 degree (blue line in Fig. 5A, long axis of the cell) and at 155 degree (green line in Fig. 5A, orthogonal to the long axis of the cell). It can be seen that all these profiles in Figs. 5B-D can be fitted with linear functions which indicate the deterministic nature of the intracellular transport [35]. The slopes of the linear functions give the bandwidths of the speed distributions, 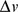 as indicated in figures. It can be seen that 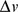 is almost double along the long axis (1.33 µm/s) of the cell compared to its orthogonal direction (0.68 µm/s) which implies that the mass transport contains faster components along the length of the cell.

**Figure 6:**
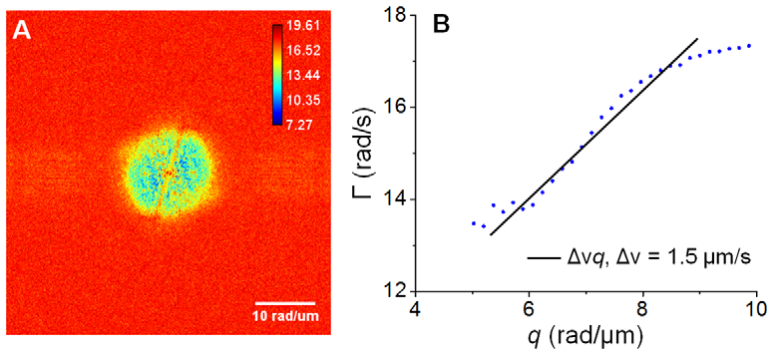
Dispersion-relation phase spectroscopy of cardiomyocyte cell 2. **A**) Dispersion relation map, Γ(*q*) associated with the cardiomyocyte cell shown in Fig. 3, **B**) Azimuthal average of data in A. The fit with the linear function yields the value of 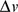 as indicated. The color bar shows Γ values in rad/s.

Figure 6 shows the DPS results for the cell shown in Fig. 3. Figure 6A shows the dispersion-relation map for this cell. Note that this cell has entirely different size and shape. Figure 6B shows the profile of the azimuthal average of data in Fig. 6A. Remarkably, this trend is again linear and the slope resulting from the fit is 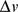 = 1.5 µm/s, which is comparable with the value obtained in Fig. 5B. Such similar values for the velocity distribution width suggest that the underlying active process driving the cell motion takes place at a universal transport velocity. Of course, before concluding on this issue, many more studies are required, which are currently ongoing in our laboratory. However, it is conceivable that having similar velocity distributions in all cells in a population encourages synchrony across the cells. By contrast, large mismatches of displacement velocities will amount to a sort of “impedance mismatch”, which will likely prevent suitable communication among the cells. Further, in Ref. [34], [35] we extracted 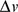 values that were much smaller (of the order of nm/sec), while the current values are around 1.5 µm/sec in a similar “q window”. Thus, it is clear that the similar values 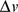 represent the physical reality of the cell transport and are not due to lack of dynamic range of our method. It is noteworthy to mention that Fig. 5A and 6A represent dispersion maps, i.e., bandwidth values at each *q*, and not just Fourier transforms of images. Thus the dispersion maps may have some numerical artifact stripes even though they may not appear in the Fourier transforms. In other words, if the noise over many Fourier transformed frames has different bandwidth values with respect to the background, the stripes will appear more significantly in the dispersion map. In Fig. 5A the noise in the horizontal direction has narrower spectrum than in the vertical direction. By contrast, Fig. 6A shows that the bandwidth noise is comparable to the background in both directions. Moreover, these artifacts occur at *q*-values higher than 12 rad which is beyond the system resolution. In order to avoid confusion, we ignore them in our analysis and plot Γ vs *q* only for *q*<*q*_0_.

## Summary and Discussion

In summary, we introduced a new instrument that promises to open new areas of study in label free cell imaging. The real-time SLIM system provides spatially and temporally resolved data describing the mass density distribution of live cells. For the first time, we were able to resolve the fast dynamics of cardiomyocytes, which allowed us to apply the DPS analysis and acquire basic knowledge about the physical nature (diffusive vs. deterministic) of cell mass displacements. We found that, over our spatiotemporal range of interest, the transport is deterministic, as one might expect from system that oscillates. Interestingly, we found that the values of the velocity distribution width are similar for cells that are completely different in size and shape. We note that the velocities measured in cardiomyocytes are 2–3 orders of magnitude larger than what we have measured previously in neurons and glial cells [12], [13]. Of course, these high velocities are due to the fast (∼2.5 Hz) beating of the cells, which induces mass displacement at the cellular scale. We anticipate that this type of investigation will teach us new aspects of intracellular and intercellular interactions.

## Supporting Information

Video S1 Video of time-lapse SLIM images of the cardiomyocyte cell shown in Fig. 4. The color bar shows the phase in radians.
(AVI)
